# Heat Stress in the Liver of Chicken: Insights from Keap1-Nrf2 Pathway Mediated Ferroptosis and Cuproptosis via the HO-1/FDX1/Gpx4 Axis

**DOI:** 10.3390/vetsci13060512

**Published:** 2026-05-26

**Authors:** Guangqing Xu, Deqiang Yan, Zekai Wang, Jinxue Ding, Yongjie Xiong, Shaojun He, Feiyang Ma

**Affiliations:** Anhui Province Key Laboratory of Animal Nutrition Regulation and Health, Anhui Science and Technology University, Chuzhou 233100, China; yjs2024410@ahstu.edu.cn (G.X.); yjs2024411@ahstu.edu.cn (D.Y.); yjs2025478@ahstu.edu.cn (Z.W.); dingjinxue@ahstu.edu.cn (J.D.); xiongyj@ahstu.edu.cn (Y.X.); hesj@ahstu.edu.cn (S.H.)

**Keywords:** heat stress, cuproptosis, ferroptosis, HO-1/FDX1/Gpx4 axis, liver

## Abstract

As the global average temperature increases, heat stress (HS) caused by high temperatures has become a key constraint to the development of the poultry industry. As the primary metabolic organ, HS can induce liver injury in chickens, thereby compromising feed safety. However, the precise mechanisms underlying HS-induced liver injury remain to be elucidated. The objective of this study is to explore the impact of HS on liver damage, oxidative stress, the Keap1-Nrf2 pathway, ferroptosis and cuproptosis in chickens. A total of 70 chickens were selected for this experiment and divided into a CON group and a HS group: the CON group was reared in a normal-temperature environment (24 ± 1°C), whilst the chronic HS group was reared in a high-temperature environment (33 ± 1°C). The findings of the study suggested that HS has the potential to induce liver dysfunction, oxidative stress, and disruption of the Keap1-Nrf2 pathway. HS has been demonstrated to induce Fe^2+^ accumulation in chicken livers, inhibit the expression of FTH1, FSP1, SLC7A11 and Gpx4, and simultaneously upregulate the expression of CD71, PTGS2 and ACSL4, thereby promoting ferroptosis. Furthermore, Cu^2+^ accumulation in the liver upregulates HSP70, DLAT and Lip-DLAT levels and downregulates the expression of ATP7B, PDH1A, PDHB, PDK4, DLST and FDX1, thereby inducing cuproptosis. Subsequent correlation analysis revealed that HS can induce ferroptosis and cuproptosis via the HO-1/FDX1/Gpx4 pathway. This finding provides new insights into the mechanisms underlying HS-induced liver injury.

## Simple Summary

Heat stress (HS) can have a negative effect on the broiler industry. HS causes significant economic losses by damaging the liver and increasing oxidative stress in chickens. Furthermore, HS can disrupt ion levels in the blood and liver tissue, leading to the occurrence of hepatic ferroptosis and cuproptosis. This study not only reveals, at the molecular level, how HS causes liver cell damage through pathways such as oxidative stress, ferroptosis and cuproptosis, but also deepens our scientific understanding of the mechanisms underlying environment–organ interactions. Furthermore, it establishes precise nutritional targets for the modern poultry industry, effectively reducing growth retardation, increased mortality, and economic losses caused by HS. It also provides crucial theoretical support for maintaining healthy production in the face of global warming.

## 1. Introduction

Elevated ambient temperatures can result in heat stress (HS) in animals, which has become a significant challenge for the global livestock industry [1]. HS can act as a significant environmental stressor, negatively affecting the performance of broiler chickens and resulting in economic losses for the poultry industry. Meanwhile, HS can cause a range of diseases in animals, including liver damage, heart damage, intestinal damage, and immune organ damage [2]. It is imperative to acknowledge that HS has been evidenced to induce liver damage, culminating in liver dysfunction. Furthermore, the results of research undertaken to date have demonstrated that HS has the potential to induced mitochondrial dysfunction in the liver [3]. Nevertheless, further comprehensive investigation is required to illuminate the molecular mechanisms by which HS instigates liver injury.

Oxidative stress is defined as a condition in which the balance between oxidation and antioxidant processes in the body is disrupted, exceeding the body’s antioxidant defence capacity and thereby leading to cellular damage [4,5]. Excessive accumulation of oxidative stress has been proven to result in the damage of proteins, DNA and organelles. It has been revealed through further research that the presence of oxidative stress within cells has the capacity to result in the disruption of mitochondrial structure and function [6]. Concurrently, the Keap1-Nrf2 pathway functions as a pivotal regulatory mechanism for oxidative stress. In recent years, research has shown that cell damage can cause different types of cell death, including apoptosis, necrosis and pyroptosis. Recent research findings indicate that cuproptosis plays a substantial role in the process of cell death [7,8]. Currently, cuproptosis plays a significant role in a wide range of diseases. Research indicates that HS exacerbates oxidative stress, increasing the demand for copper-dependent antioxidant enzymes (such as Cu/Zn-SOD), which leads to improved copper utilization in the body [9]. What is more, copper can stick to dihydrolipoamide S-acetyltransferase (DLAT) and encourage it to be lipoylated, which can trigger cuproptosis [10,11]. Research has shown that cuproptosis is involved in a variety of diseases, but we still do not know what its role is in HS.

Ferroptosis is defined as a form of programmed cell death that is dependent on iron and is defined by the progressive accumulation of lipid peroxides, which leads to the disruption of membrane structure by oxidation [12]. As a pivotal molecule in ferroptosis, glutathione peroxidase 4 (Gpx4) effectively maintains cellular lipid homeostasis and prevents the onset of ferroptosis. Concurrently, under the influence of copper ions, Gpx4 is more readily degraded via the autophagy pathway, thereby indirectly promoting iron-induced ferroptosis [13,14]. This finding underscores a pivotal nexus between cuproptosis and ferroptosis, thereby imparting novel insights into the mechanisms of cellular damage engendered by the disruption of metal ion homeostasis. As evidenced by Zhang et al. [15], ferroptosis has been shown to play a pivotal role in hepatocyte injury triggered by HS. Concurrently, copper-binding ferredoxin 1 (FDX1), a pivotal regulator of cuproptosis, functions in both iron metabolism and transport [16]. Despite the differences in their mechanisms, ferroptosis and cuproptosis are both characterized by dysregulation of metal ion metabolism and elevated oxidative stress. This finding indicated the potential for cross-regulatory interactions under specific physiological conditions. Consequently, ascertaining the mechanisms of HS-induced liver injury and the interplay between cuproptosis and ferroptosis is of significant importance.

In accordance with the aforementioned fundamental scientific inquiries, this study employed AA broiler chickens as experimental subjects for the purpose of establishing an HS model. The present study is chiefly concerned with the molecular mechanisms underlying HS-induced liver injury and with elucidating the potentially interactive relationship between cuproptosis and ferroptosis. This research offers significant scientific substantiation for the formulation of preventative and therapeutic measures for HS, thus facilitating the mitigation of economic losses.

## 2. Materials and Methods

### 2.1. Animals and Treatment

This animal experiment was approved by the Animal Experiment Management Committee of the Anhui Science and Technology University (Approval No. AK2025080). Seventy 1-day-old healthy Arober Acres (AA) broiler chicks were selected for the experiment and fed (Table S1) and watered ad libitum during the feeding period. After feeding until 28 days of age, they were divided into a control group (CON) (environment temperature 24  ±  1 °C) and a heat stress group (HS) (environment temperature 33  ±  1°C) [17,18]. Relative humidity was kept at 60 ± 5%. At 42 days of age, animals in each group were euthanized under ether anesthesia, and serum and liver were collected.

### 2.2. Biochemical Analysis

After blood collection, the serum was separated. The concentrations of lactate dehydrogenase (LDH), aspartate aminotransferase (AST), alanine aminotransferase (ALT), and alkaline phosphatase (ALP) in the serum were then analyzed using an automated blood chemistry analyzer (Mindray BS-200, Shenzhen, China).

### 2.3. Histopathological Analysis

Chicken liver was fixed in 4% paraformaldehyde solution; gradient xylene and alcohol treatment were performed as per the previously described method. HE staining of the liver was performed using hematoxylin–eosin staining. Liver Masson and PAS staining were performed according to the previously described method, and the results were observed by microscopy.

### 2.4. Determination of Oxidative Stress Level

Weigh out 100 mg of liver tissue and add 900 µL of saline solution, then mix it all together. Centrifuge at 3500 rpm for 15 min, then collect the supernatant. Determine total antioxidant capacity (T-AOC), glutathione peroxidase (GSH-Px), superoxide dismutase (SOD) and malondialdehyde (MDA) levels according to the kit instructions [18]. Concurrently, the protein concentration in liver tissue is measured, and the levels of relevant markers are calculated.

### 2.5. Determination of Cu and Iron Levels

Take an appropriate amount of serum and liver samples. The Cu^2+^, Fe^2+^ and total iron assays should be carried out in conformity with the kit instructions [19].

### 2.6. Immunohistochemical Assay

Liver sections were subjected to a deparaffinization process using xylene, following a standard protocol, followed by antigen retrieval using citrate buffer. Subsequently, the sections were immersed in a blocking solution comprising 10% horse serum. Subsequent to this, the incubation process was continued for a period of 16 h, during which the following antibodies were utilized: CD71 (1:1000, Servicebio, Wuhan, China), PTGS2 (1:200, ABclonal, Wuhan, China) and ATP7B (1:200, Zenbio, Chengdu, China). The results obtained are presented in photographs taken after staining with DAB and hematoxylin.

### 2.7. Immunofluorescence Assay

The pre-treatment was analogous to immunohistochemistry, followed by cultivation with 8-OHdg, DLAT (1:50, ABclonal, Wuhan, China), HO-1 (1:100, Proteintech, Wuhan, China), Gpx4 (1:200, Proteintech, Wuhan, China), and FDX1 (1:200, ABclonal, Wuhan, China) antibodies, with a duration of 14 h. DAPI staining (Beyotime, Shanghai, China) was then conducted, followed by a secondary antibody incubation. The findings were meticulously recorded through the utilization of confocal microscopy (Leica, Wetzlar, Germany).

### 2.8. Western Blot Analysis

The Western blotting assay protocol was consistent with the established method [4,7]. The primary antibody is shown in Table S2. Finally, images were captured following ECL development, and the grayscale values were standardized and analyzed using ImageJ (version 1, National Institutes of Health, Bethesda, MD, USA).

### 2.9. Molecular Docking

The investigation of potential interactions between target proteins is facilitated by the utilization of molecular docking techniques. The protein structures were predicted using AlphaFold 3 and visualized using PyMOL (version 2.5.4, Schrödinger, LLC, New York, NY, USA) Subsequently, molecular docking was undertaken using AutoDock Vina (version 1.2.5, The Scripps Research Institute, La Jolla, CA, USA) and the results were rendered in PyMOL.

### 2.10. Statistical Analysis

The data were examined using SPSS version 24.0 (IBM Corp., Armonk, NY, USA) and presented as the mean ± standard deviation (SEM) (n = 6). The generation of statistical graphs was facilitated by the GraphPad Prism 8.0 software programme (GraphPad Inc., La Jolla, CA, USA). The confidence intervals were calculated as follows: * for *p* < 0.05, ** for *p* < 0.01, and *** for *p* < 0.001.

## 3. Results

### 3.1. Heat Stress-Induced Liver Injury in Chicken

**Figure 1:**
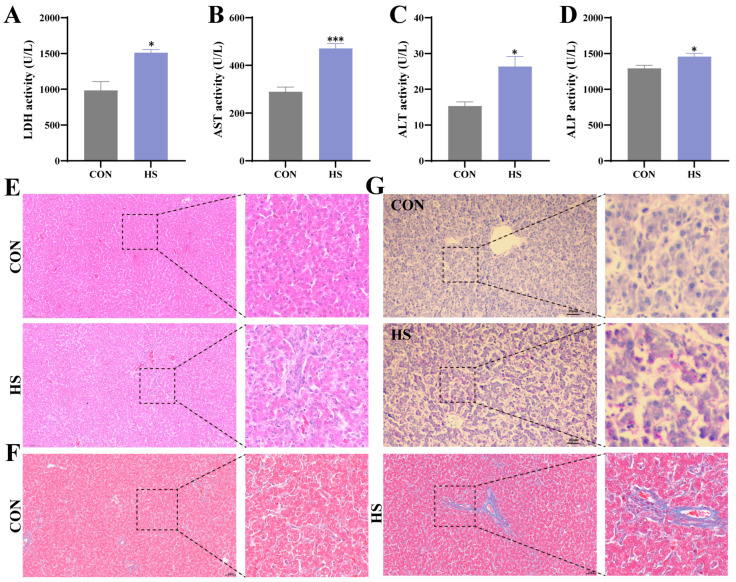
Heat stress-induced liver injury. (**A**) LDH level. (**B**) AST level. (**C**) ALT level. (**D**) ALP level. (**E**) HE staining results. (**F**) Observations on Masson staining. (**G**) PAS staining results. All data were expressed as mean ± SEM; “*” indicates statistically significant difference with the CON group (* *p* < 0.05, *** *p* < 0.01).

We investigated the effects of HS on the liver. Serum biochemical markers (LDH, AST, ALT, ALP) were utilized to evaluate liver function. The findings indicated that, compared with the CON group, HS led to a substantial augmentation in the levels of these markers (*p* < 0.05) (Figure 1A–D). In contrast, the hepatic lobules in the HS group exhibited a disorganized pattern, accompanied by the presence of inflammatory cell infiltration. It is also worth noting that mild degenerative changes in hepatocytes were also identified in the liver of the HS group (Figure 1E). Concurrently, Masson staining revealed increased collagen fibre deposition (blue) in the HS group compared with the CON group (Figure 1F). PAS staining revealed increased glycogen deposition (purple) in the HS group compared with the CON group (Figure 1G). The results of the present study demonstrate that HS can induce liver damage in chickens.

### 3.2. Effect of Heat Stress on Oxidative Stress in Chicken Liver

**Figure 2:**
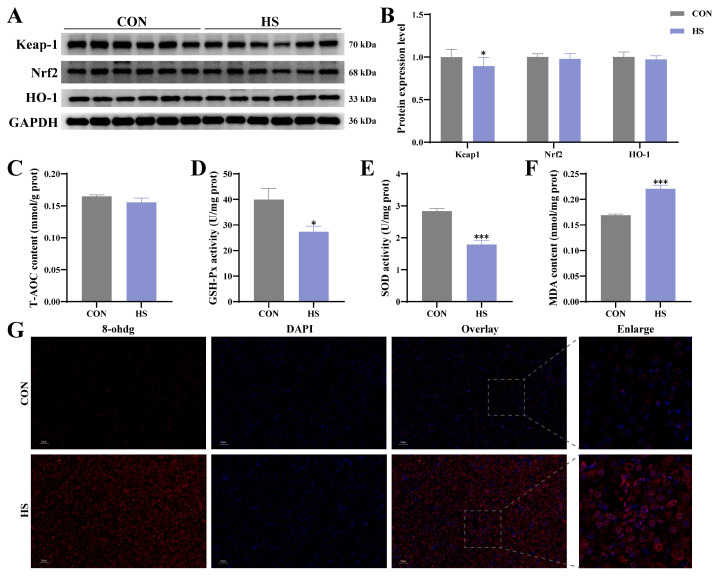
Heat stress-induced oxidative stress in the liver. (**A**) Protein results graph. (**B**) Analysis of protein results. (**C**) T-AOC level. (**D**) GSH-Px level. (**E**) SOD level. (**F**) MDA level. (**G**) 8-ohdg immunofluorescence results. All data were expressed as mean ± SEM; “*” indicates statistically significant difference with the CON group (* *p* < 0.05, *** *p* < 0.01).

This study examines the effect of HS on oxidative stress in the liver through the Keap1-Nrf2 pathway. The results of protein imaging and analysis demonstrated that the levels of proteins Keap1, Nrf2, and HO-1 were reduced in the HS group, but these changes were not deemed to be significant (Figure 2A,B). Subsequent analysis of antioxidant markers revealed no significant alteration in the levels of T-AOC in the liver (Figure 2C). Concurrently, a marked decline in the enzymatic activities of GSH-Px and SOD was observed in the HS group when compared with the CON group (*p* < 0.05) (Figure 2D,E). In addition, a significant increase in MDA levels was detected in the HS group compared with the CON group (*p* < 0.05) (Figure 2F). 8-ohdg, a significant biomarker of oxidative DNA damage, exhibited a substantial increase in the HS group, as evidenced by immunofluorescence analysis (Figure 2G). The above findings indicate that HS can induce liver damage.

### 3.3. Effect of Heat Stress on Ferroptosis in Chicken Liver

**Figure 3:**
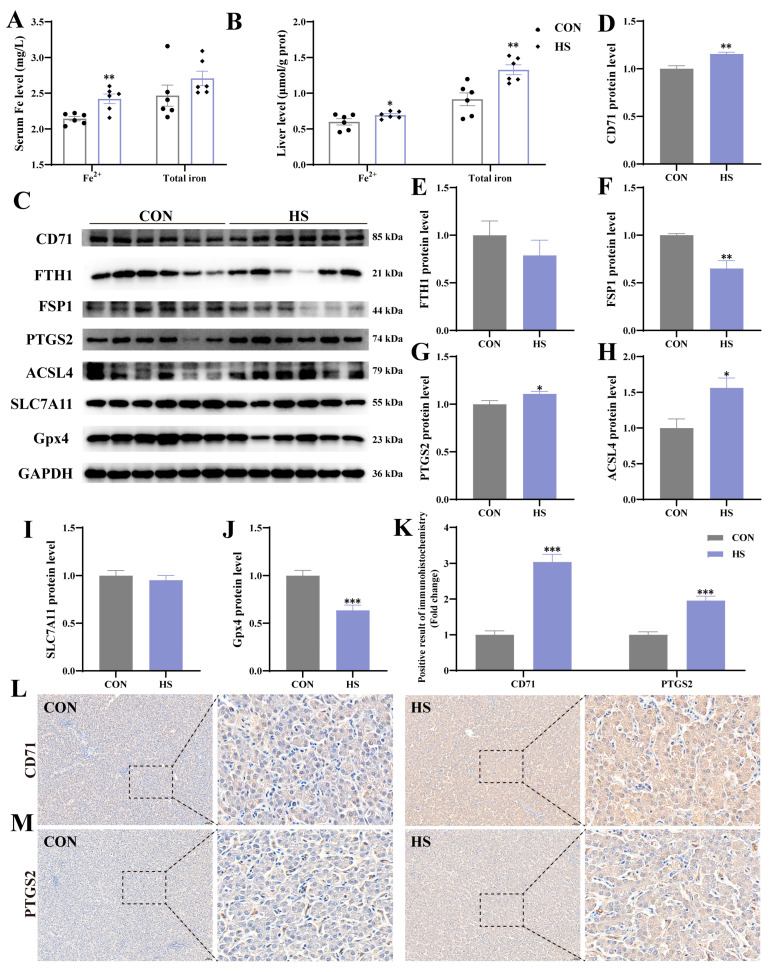
Heat stress-induced ferroptosis in the liver. (**A**) Serum Fe^2+^ and total iron levels. (**B**) Liver Fe^2+^ and total iron levels. (**C**) Protein results graph. (**D**–**J**) Analysis of protein results. (**K**–**M**) Immunohistochemical results and analysis for CD71 and PTGS2. All data were expressed as mean ± SEM; “*” indicates statistically significant difference with the CON group (* *p* < 0.05, ** *p* < 0.01, *** *p* < 0.01).

The current study explores the effect of HS on ferroptosis in the liver. Serological analysis indicated that Fe^2+^ levels were heightened in the HS group (*p* < 0.05), while total iron levels remained unchanged (Figure 3A). Concurrently, levels of Fe^2+^ and total iron in the liver were elevated in the HS group compared with the CON group (*p* < 0.05) (Figure 3B). The results for ferroptosis-related proteins are shown in Figure 3C. Protein analysis revealed that, compared with the CON group, the levels of CD71, PTGS2, and ACSL4 were higher in the HS group (*p* < 0.05) (Figure 3D,F–H). However, no significant alterations in the levels of FTH1 and SLC7A11 were observed (Figure 3E,I). Concurrently, the levels of FSP1 and Gpx4 were found to be decreased in the HS group compared to the CON group (*p* < 0.05) (Figure 3J). The results of the immunohistochemical tests showed an increase in the levels of proteins CD71 and PTGS2 in the HS group compared to the CON group (*p* < 0.05) (Figure 3K–M). The findings outlined above suggest that HS can induce ferroptosis in chicken livers.

### 3.4. Effect of Heat Stress on Cuproptosis in Chicken Liver

**Figure 4:**
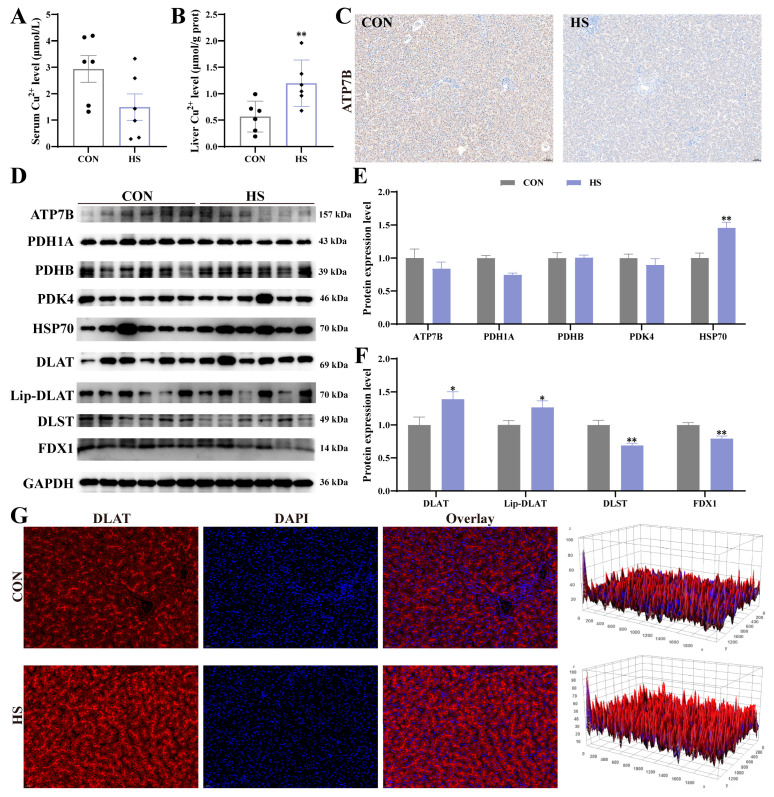
Heat stress-induced cuproptosis in the liver. (**A**) Serum Cu^2+^ levels. (**B**) Liver Cu^2+^ levels. (**C**) Immunohistochemical results of ATP7B. (**D**) Protein results graph. (**E**,**F**) Analysis of protein results. (**G**) DLAT immunofluorescence results and analysis. All data were expressed as mean ± SEM; “*” indicates statistically significant difference with the CON group (* *p* < 0.05, ** *p* < 0.01).

The serological analysis demonstrated that the HS group exhibited reduced levels of Cu^2+^ compared with the CON group (Figure 4A). Concurrently, a significant elevation in liver Cu^2+^ levels was observed in the HS group compared with the CON group (*p* < 0.05) (Figure 3B). The results of the immunohistochemical analysis demonstrated that, compared with the CON group, the level of ATP7B-brown positivity was reduced in the HS group (Figure 4C). The results pertaining to cuproptosis-related proteins are illustrated in Figure 4D. The analysis of the protein results indicates a downward trend in the levels of proteins ATP7B, PDH1A, PDHB, PDK4, DLST and FDX1 in the HS group (Figure 4E,F). Concurrently, proteins HSP70, DLAT and Lip-DLAT exhibited a marked increase in the HS group compared with the CON group (*p* < 0.05). The immunofluorescence results indicated a marked increase in the fluorescence intensity of DLAT in the HS group compared to the CON group (Figure 4G). The findings outlined above suggested that HS can induce cuproptosis in chicken livers.

### 3.5. Correlation Analysis of HO-1, FDX1 and Gpx4

**Figure 5:**
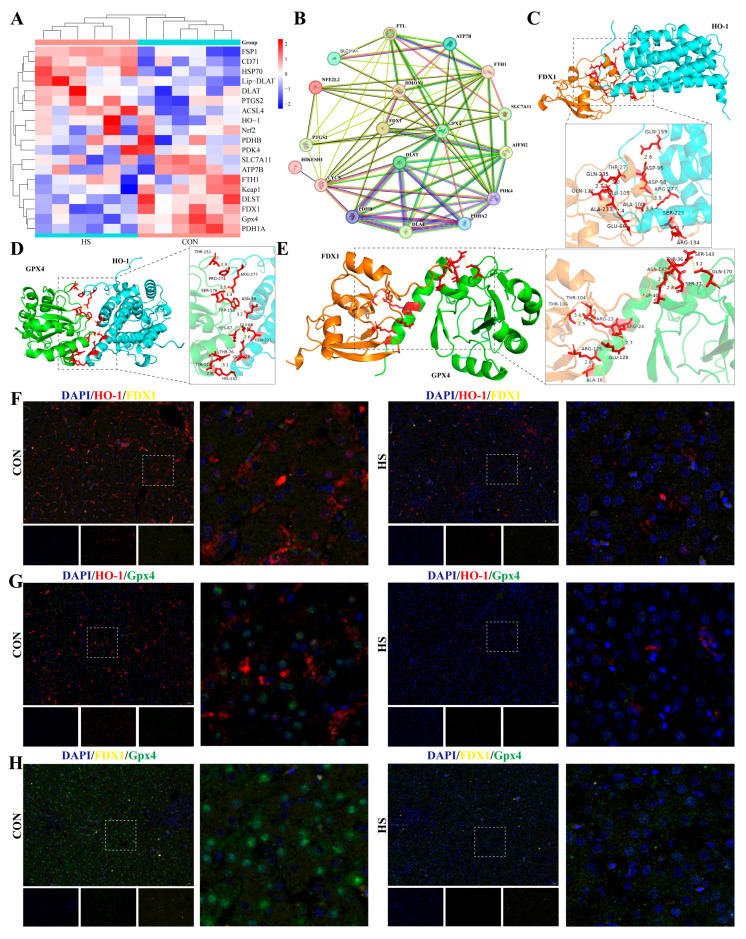
Protein correlation analysis. (**A**) Protein interaction heatmap. (**B**) Analysis of PPI correlations. (**C**) Molecular docking results of HO-1 and FDX1 molecules. (**D**) Molecular docking results of HO-1 and Gpx4 molecules. (**E**) Molecular docking results of FDX1 and Gpx4 molecules. (**F**–**H**) Immunofluorescence results for HO-1, Gpx4 and FDX1.

In light of the aforementioned findings, it is hypothesized that there may be a correlation between the Keap1-Nrf2 signalling pathway and the processes of iron-mediated ferroptosis and copper-mediated cuproptosis (Figure 5A). STING predictions revealed a relationship among HO-1, FDX1 and Gpx4 (Figure 5B). Furthermore, to investigate this issue, we first used AlphaFold3 to predict the three-dimensional structures of chicken HO-1, FDX1 and Gpx4. Molecular docking revealed interactions among them (Figure 5C–E). To gain a more comprehensive and intuitive insight into these alterations, the fluorescent localization of HO-1, FDX1 and Gpx4 was examined further using immunofluorescence confocal microscopy (Figure 5F–H). The results indicated that, under the influence of HS, the number of fluorescent levels for HO-1, FDX1 and Gpx4 was significantly reduced.

## 4. Discussion

In the context of global environmental changes, HS has emerged as a substantial health hazard for animals, particularly given its capacity to induce organ damage in poultry [20]. HS has been demonstrated to cause a disruption in liver function and metabolism, with the potential to impair multi-organ function, thus constituting a grave threat to animal and human health [21]. HS causes tissue injury in organisms and disrupts the body’s oxidative balance. Moreover, the prevailing consensus among numerous scholars is that the mechanisms underlying HS injury may encompass multiple cell death phenomena. However, the relationship between ferroptosis and cuproptosis in HS-induced hepatic injury remains incompletely elucidated. The present study employed histopathological observation to identify alterations in hepatic function and pathology. Subsequent research has indicated that disruption of copper and iron metabolism in the livers of HS broiler chickens can be facilitated by the Keap1-Nrf2 pathway, resulting in ferroptosis and cuproptosis.

It is widely acknowledged that the deleterious effects of HS are associated with the generation of reactive oxygen species (ROS), which in turn leads to oxidative damage to organisms [22]. The Keap1-Nrf2 pathway has been identified as a pivotal regulatory mechanism in cellular responses to oxidative stress [23]. In typical circumstances, Nrf2 is bound by its repressor protein, Keap1, and targeted for ubiquitination and degradation. This process ensures the maintenance of stable expression levels [24]. During periods of oxidative stress, this pathway activates the transcription of multiple antioxidant enzyme genes. Superoxide dismutase (SOD) and glutathione peroxidase (GSH-Px) are essential antioxidant enzymes that protect cells by eliminating reactive oxygen species and breaking down peroxides, while total antioxidant capacity (T-AOC) reflects the body’s overall ability to counteract oxidative stress [25]. Conversely, malondialdehyde (MDA), a by-product of lipid peroxidation, functions as a pivotal indicator of oxidative damage. The findings of this study are corroborated by those of preceding research [26,27,28], suggesting that HS functions as a mediator for oxidative stress in the liver via the Keap1-Nrf2 pathway. Meanwhile, 8-hydroxy-deoxyguanosine (8-ohdg) is an oxidation product of deoxyguanosine and an established biomarker of oxidative DNA damage [29]. This study found that immunofluorescence results showed increased levels of 8-OHdG fluorescence in the cytoplasm of hepatocytes in the HS group, indicating mitochondrial DNA damage. Furthermore, as the cell’s powerhouse, mitochondria fulfil a number of functions. These include the regulation of cell proliferation, apoptosis and signal transduction [30]. The findings outlined above indicate that HS has the capacity to induce Keap1-Nrf2-mediated oxidative stress and mitochondrial damage within the liver.

HS can induce oxidative stress and mitochondrial damage in hepatocytes, thereby exacerbating metabolic dysfunction. At the same time, the imbalance of trace elements within the body caused by HS has been extensively studied, and can lead to disturbances in hepatic iron metabolism [31]. Consequently, the absorption and transport of iron in the liver is disrupted by HS, which may lead to pathological iron accumulation. HS has been demonstrated to disrupt iron metabolism in the liver, with this disruption potentially inducing ferroptosis [32]. Ferroptosis is characterized by a dynamic equilibrium between iron uptake, facilitated by CD71, and iron storage, driven by FTH1 (ferritin heavy chain 1) [33]. Disruption of this balance exerts a substantial influence on cellular fate. In addition, as a glutathione-independent ferroptosis inhibitor, FSP1 functions as a redox enzyme, thereby inhibiting lipid peroxidation [34]. Furthermore, ACSL4 has been proven to instigate ferroptosis by promoting the transfer of polyunsaturated fatty acids to membrane phospholipids, thereby rendering the membrane phospholipids more susceptible to peroxidation. PTGS2 has been demonstrated to be intimately associated with the ferroptosis process and is extensively utilized as a molecular marker for ferroptosis [35]. Gpx4 primarily exerts its protective effects by scavenging lipid peroxides, whilst SLC7A11 facilitates cystine uptake to support glutathione synthesis, thereby providing the functional basis for Gpx4 [36]. It has been demonstrated by related studies that exposure of broiler chicken livers to HS can result in the induction of ferroptosis [37,38]. This study found that HS increased total iron and Fe^2+^ levels in serum and the liver, while reducing the liver’s FTH1 protein expression levels. Furthermore, HS resulted in significant upregulation of CD71, PTGS2 and ACSL4 protein levels, and downregulation of FSP1, SLC7A11 and Gpx4 protein levels in the liver. These results imply that HS induces ferroptosis in the liver due to iron overload.

Cuproptosis, a recently identified form of cell death, is initiated by the excessive accumulation of copper ions. This results in the aberrant accumulation of thiol-modified proteins, the disruption of iron–sulphur proteins involved in mitochondrial respiration, and the subsequent occurrence of protein-induced toxic stress-mediated cell death [10]. The findings of the research suggested that copper accumulation has the potential to induce cuproptosis [19]. The present study suggests that, in HS chickens, serum copper levels were reduced, while liver copper levels were significantly elevated. This phenomenon may be attributed to the elevated levels of inflammatory cytokines and acute-phase proteins, which are known to induce a heightened transfer of copper from the blood to the liver [9]. Concurrently, the interaction of acetylated DLAT with copper has been demonstrated to result in its aberrant aggregation, thereby instigating copper-induced cuproptosis. This is similar to other studies that have shown that, in heart failure, an imbalance of copper metabolism can trigger a process called cuproptosis via the SIRT1/HMGB1 pathway [39]. The experimental results obtained in this study suggest that HS can induce cuproptosis by regulating key proteins, including ATP7B, PDHA1, PDHB, PDK4, HSP70, DLAT, Lip-DLAT and FDX1. The current study’s findings indicate that HS can lead to the accumulation of copper within the liver and the induction of cuproptosis.

Cuproptosis is characterized by a distinct molecular mechanism whilst also exhibiting certain similarities to the molecular mechanism of ferroptosis [40]. As a key regulator of ferroptosis, Gpx4 has been demonstrated to bind directly with copper ions, inducing their aggregation and subsequent degradation via the autophagic pathway, thereby diminishing their capacity to inhibit cuproptosis [41]. FDX1 is a small-molecule protein that comprises an iron–sulphur centre and functions in a variety of essential biological processes. Moreover, any disruption to the biosynthesis of iron–sulphur clusters has the potential to disrupt intracellular iron homeostasis and mitochondrial function, thereby exacerbating the occurrence of ferroptosis [42]. In this study, it was observed that HS upregulates the acetylation levels of DLAT. This finding suggests the potential for a degree of mutual regulation and an underlying mechanism linking cuproptosis and ferroptosis. The experimental data obtained in the present analysis suggest a considerable decline in the levels of FDX1 and Gpx4, accompanied by a decrease in their fluorescent colocalization, in response to HS. In addition, the prediction of the protein configurations of FDX1 and Gpx4 has revealed an interaction between these proteins, at least to a certain extent, with respect to their structural configuration. The findings of this study indicate that the interaction between HS-induced cuproptosis and ferroptosis may be facilitated by the interplay between FDX1 and Gpx4. Furthermore, STING results and molecular docking studies indicate an interaction of HO-1, FDX1 and Gpx4. Research findings indicate a correlation between HO-1 and cuproptosis [43,44]. Concurrently, HO-1 exerts a substantial influence on the process of ferroptosis [45,46]. The present study indicates that the HO-1/FDX1/Gpx4 axis is pivotal in the correlation between HS-induced cuproptosis and ferroptosis.

## 5. Conclusions

In conclusion, the results of this study indicate that exposure of chicken liver to HS leads to an imbalance of trace elements, which in turn promotes cuproptosis and ferroptosis via the HO-1/FDX1/Gpx4 axis.

## Data Availability

The original contributions presented in this study are included in the article/Supplementary Material. Further inquiries can be directed to the corresponding author.

## Supplementary Materials

The following supporting information can be downloaded at https://www.mdpi.com/article/10.3390/vetsci13060512/s1, Table S1: The composition and nutrients levels in the diet for chicken; Table S2: Antibodies of relevance in the study; Figures: Original Western blot images.
