# Supplementary material for: Heat Stress in the Liver of Chicken: Insights from Keap1-Nrf2 Pathway Mediated Ferroptosis and Cuproptosis via the HO-1/FDX1/Gpx4 Axis
Source: Vet Sci. 2026 May 26;13(6):512. doi: 10.3390/vetsci13060512 (PMC13307753; doi:10.3390/vetsci13060512)
Supplement: Supplementary file 1 [file vetsci-13-00512-s001.zip › Supplementary material-.pdf]

Supplementary material

Supplementary table

Table S1 The composition and nutrients levels in the diet for chicken.

Composition of diet		Nutrients levels	
Ingredient	Content (%)	Index	Level
Maize	54.82	DM (%)	88.6
Soybean meal	37.77	CP (%)	23
Soybean oil	2.5	ME (MJ kg^{-1})	12.7
Limestone	1.01	Ca (%)	0.95
$CaHPO_4$	1.75	AP (%)	0.46
Salt	0.4		
DL-methionine	0.25		
Corn starch + Cu	1		
Additives*	0.5		
Total	100		

* Per kilogram of additives contained the following: Vitamin A 6600 IU, Vitamin D 2200 IU, Vitamin K_1 2.2 mg, Vitamin B_2 4.4 mg, pantothenic acid 13 mg, nicotinic acid 40 mg, choline chloride 500 mg, Vitamin B_{12} 22 µg, Fe ($FeSO_4 \cdot H_2O$) 500 mg, Mn ($MnSO_4 \cdot H_2O$) 60 mg, Se ($NaSe_3O_4$, 1%) 0.2 mg, I (KI, 3%) 0.35 mg.

Table S2. Antibodies of relevance in the study.

Antibody	Host	Product Number	Brand
GAPDH	Mouse	60004-1-Ig	Proteintech, China
Keap-1	Rabbit	10503-2-AP	Proteintech, China
Nrf2	Rabbit	16396-1-AP	Proteintech, China
HO-1	Rabbit	10701-1-AP	Proteintech, China
CD71	Mouse	GB150088	Servicebio, China
FTH1	Rabbit	A19544	ABclonal, China
FSP1	Rabbit	A22278	ABclonal, China
PTGS2	Rabbit	A3560	ABclonal, China

ACSL4	Rabbit	A20414	ABclonal, China
SLC7A11	Rabbit	A2413	ABclonal, China
Gpx4	Mouse	67763-1-Ig	Proteintech, China
ATP7B	Rabbit	R389236	Zenbio, China
PDH1A	Mouse	200270	Zenbio, China
PDHB	Rabbit	R382198	Zenbio, China
PDK4	Rabbit	12949-1-AP	Proteintech, China
HSP70	Mouse	R24633	Zenbio, China
DLAT	Rabbit	A14530	ABclonal, China
Lip-DLAT	Rabbit	ab58724	Abcam, China
DLST	Rabbit	A13297	ABclonal, China
FDX1	Rabbit	A9815	ABclonal, China
