# Supplementary figures and images for: Heat Stress in the Liver of Chicken: Insights from Keap1-Nrf2 Pathway Mediated Ferroptosis and Cuproptosis via the HO-1/FDX1/Gpx4 Axis
Source: Vet Sci. 2026 May 26;13(6):512. doi: 10.3390/vetsci13060512 (PMC13307753; doi:10.3390/vetsci13060512)

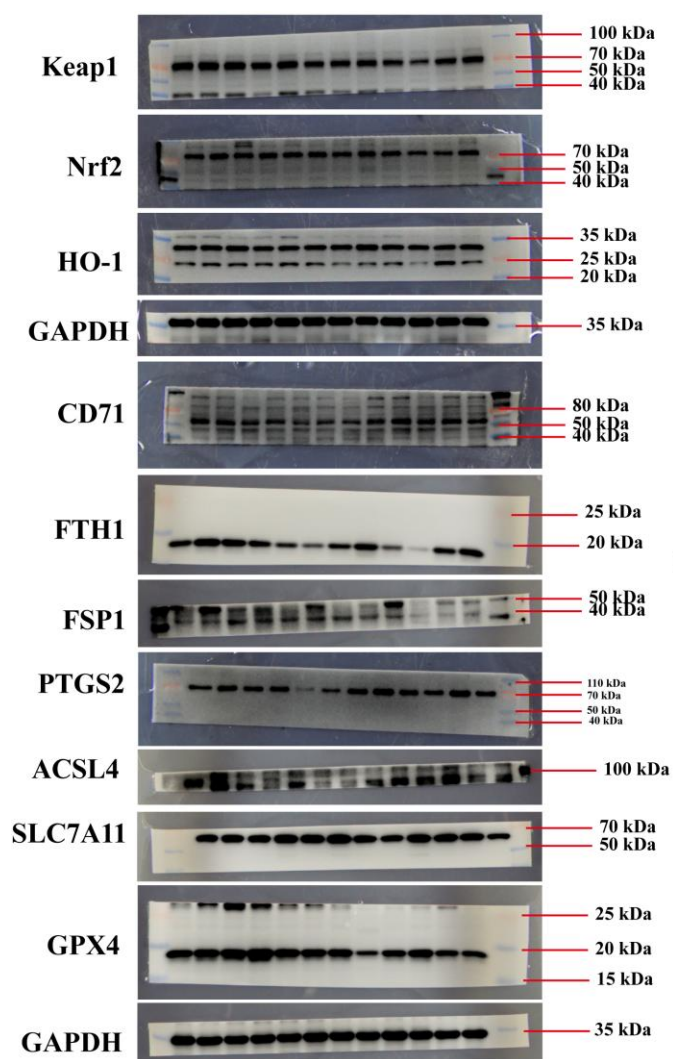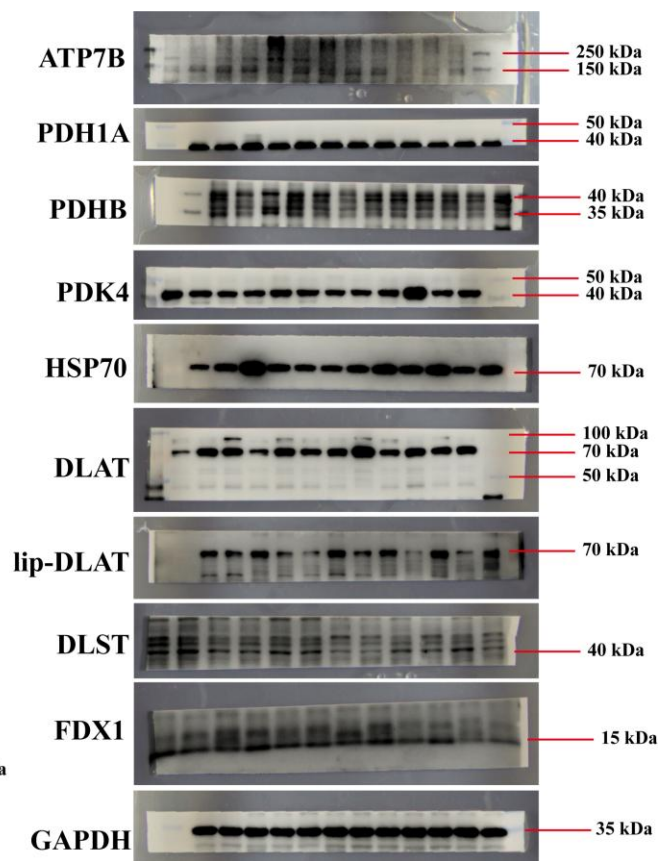

Supplement: Supplementary file 1 [file vetsci-13-00512-s001.zip › Protein result element diagram2.pdf]
